# The role of orthographic and phonological processing during reading Chinese sentences: Evidence from eye movements

**DOI:** 10.3389/fpsyg.2023.1148815

**Published:** 2023-08-17

**Authors:** Zhang Lijuan, Zhang Yingying, Liu Zhiwei, Li Lin, Li Sha, Wang Jingxin

**Affiliations:** ^1^Faculty of Psychology, Tianjin Normal University, Tianjin, China; ^2^College of Sports and Health, Shandong Sport University, Jinan, Shandong, China; ^3^School of Education and Psychology, Sichuan University of Science and Engineering, Zigong, Sichuan, China; ^4^School of Psychology, Fujian Normal University, Fuzhou, Fujian, China

**Keywords:** orthography, phonology, misspelled characters, eye movements, Chinese reading

## Abstract

The role of phonological and orthographic processing and their time course during lexical processing and sentence reading remain controversial. By adopting a misspelled-characters disruption paradigm and eye-tracking technique, we manipulated the writing for the first characters of two-character target words to investigate the relative role of orthographic and phonological processing on word recognition in Chinese reading. There are four conditions: (a) correct character, (b) misspelled character with a stroke missing, (c) misspelled homographic character, and (d) misspelled homophonic character. The results showed that homophonic errors caused more disruptions than other conditions in the early (first-pass reading times) and later (total reading time) stages of lexical processing during Chinese reading. Homographic errors and omitted stroke errors lead to equal disruptions at the early stage of word recognition, but homographic errors cause more disruptions at the later stage. These results suggest that orthography plays a dominant role in word recognition during Chinese reading, whereas phonology plays a weaker and more limited role. The direct access and dual-rote hypothesis may well explain the mechanism of lexical processing in Chinese reading.

## 1. Introduction

Reading involves extracting visual information from the page and comprehending the meaning of the text (Rayner et al., 2012). To comprehend the text or a sentence, readers need to recognize the words in it and complete lexical access, which has been considered the engine that drives eye movements in reading (Reichle et al., 1998). Skilled readers achieve lexical access by constantly collecting a large amount of lexical information during sentence reading. Previous studies have demonstrated that phonological and orthographic information can be activated before lexical access is completed (Seidenberg and McClelland, 1989; Harm and Seidenberg, 2004). However, to date, the role of phonological and orthographic processing and their time course during lexical processing and sentence reading remain controversial. Using an eye-tracking technique, the present study examined this issue in Chinese reading.

Several models for the role of phonology and orthography in lexical access during reading have been proposed. The direct access hypothesis suggests that the meaning (semantic information) of words is accessed directly through orthographic representation (based on visual input), bypassing phonological information encoding (Zhou and Marslen-Wilson, 1996; Zhou et al., 1999a; Zhang et al., 2020). The phonologically meditated access hypothesis, on the other hand, holds that visual (orthographic) information first activates phonological information and then activates semantic information; therefore, phonological information plays a dominant role in lexical processing (Frost, 1998; Perfetti and Zhang, 1995; Liu et al., 2003). However, according to the dual-rote hypothesis, both orthographic direct access and phonological mediation access exist and interact with each other during lexical access (Coltheart et al., 2001; Harm and Seidenberg, 2004). The controversy in these models lies in the relative importance of direct visual (orthographic) or phonological properties in lexical processing, whereas the ultimate goal is to access the meaning of the words and text (Li et al., 2022).

Alphabetic language studies on this issue failed to obtain consistent results in the process of reading. Several studies on word recognition or picture naming task have suggested that phonological information is activated automatically and early, and the phonological processing is independent of orthography (Frost, 1998; Kinoshita and Verdonschot, 2021). Some eye movement studies on English reading have found that phonological information could be activated before a word is fixated (Pollatsek et al., 1992; Sereno and Rayner, 1992; Vasilev et al., 2019). Other eye movement studies have also shown that skilled readers could rapidly generate phonological codes during reading, which will affect the recognition of most words (Leinenger, 2019). For example, homophones related to the target words are more easily processed. These studies suggest that phonological information is activated at a relatively early stage and plays a major role in lexical processing during English reading. However, other studies using spelling error words found that phonological information could only play a role in lexical access under certain conditions. For instance, Daneman and Reingold (1993) used an error disruption paradigm (some keywords were replaced by homophonic error words or non-homophonic but orthographically similar error words) and found that phonological effects only occurred at a later stage of lexical processing when the homophonic word was orthographically similar to the target word. This pattern of results was replicated in another related study (Daneman et al., 1995). In addition, in a recent Spanish study (Marcet and Perea, 2022), it was found that when the accent mark was removed (still orthographically similar to the target word), phonological information (e.g., accent marks in words) had no effect at early stages of word processing, as measured by the first-pass reading times, but had a sizable effect at later stages (total reading time).

Thus, for the alphabetical writing system, it is still debatable whether phonological information plays a major role in the early stage of lexical processing or plays a limited role in the later stage under certain situations. Although the meta-analysis of phonological preview benefit shows that many studies have found that English readers can extract phonological information in the early stage of lexical processing, the phonological preview benefit in English is not that strong (Vasilev et al., 2019). For languages using the Roman script (for instance, English, Spanish, and French, among others), it may be difficult to isolate phonological and orthographical effects due to strong grapheme-phoneme correspondences (the strong overlap between orthography and phonology) (Vasilev et al., 2019; Meade, 2020). Recent studies have also shown that the grapheme-phoneme pattern regulates the effect of phonology on the time course of word processing (Labusch et al., 2022). Moreover, even orthographic control condition typically shares one phoneme (at least) of the target word. This means that orthographic and phonological information is often confused in previous studies using homophones and homographs in English words.

The Chinese writing system has some unique properties that differ from the alphabetic writing system and provide some advantages for investigating this issue. Chinese characters originate from pictographs, and there is a close connection between graphic form and meaning (Tan and Perfetti, 1999). The graphic form of a character often vividly represents its meaning. Different from the alphabetical writing system, there is a weak relationship between pronunciation and orthography in the Chinse writing system. Furthermore, many Chinese characters that differ in lexical meanings have similar orthography (homographic characters) or the same pronunciation (homophonic characters) (Feng et al., 2001; Meng et al., 2008). More importantly, homographic characters often have no phonetic similarities, and homophonic characters have no orthographic similarities. For example, 项 (xiang, meaning neck) and 顶 (ding, meaning top) share similar orthographic information but differ in pronunciation. 项 (xiang, meaning neck) and 像 (xiang, meaning like) have the same pronunciation without orthographic similarity. Moreover, homographic or homophonic characters often have no semantic connections or relationships. The unique features of the Chinese writing system allow us to differentiate the role of orthographic and phonological processes in lexical processing during reading.

Because of the unique characteristics of Chinese characters, such as the high transparency of the orthography that can directly represent semantic information, phonological information may play a weaker role in lexical processing than orthography when compared to its role in alphabetic languages. Some studies have found that Chinese readers can directly access the meaning of words without going through the phonologically mediated route (Zhou and Marslen-Wilson, 1996; Zhou et al., 1999a; Zhang et al., 2020). For example, previous studies using various paradigms, such as semantic categorization (Leck et al., 1995), backward masking (Perfetti and Zhang, 1991), and lexical decisions with two-character words (Zhou et al., 1999a) have found weak or no phonologically mediated priming effects. Other research has found that even when a Chinese character’s pinyin (phonological information) is available, access to lexical representation is orthographically centered (Chen et al., 2019). In addition, electrophysiological studies have found that phonology plays a limited role in Chinese word recognition. For example, evidence from ERP studies has suggested that readers rely more on orthographic information than phonological information to access the semantics of words during Chinese reading (Meng et al., 2008; Wong et al., 2014; Zhang et al., 2020). These findings suggest that orthographic information plays a major role in lexical semantic access, supporting the direct access route.

However, different results have been obtained in other Chinese studies. Numerous studies have shown that phonological information plays a role in the early stages of word processing. For example, several Chinese studies using various paradigms have shown that phonological information can be activated early in lexical access (Perfetti and Zhang, 1995; Tan et al., 1995). However, most research demonstrating early activation of phonological information is difficult to replicate (Chen and Shu, 2001). In addition, the meta-analysis of Vasilev et al. (2019) also found that there is a phonological preview benefit in Chinese reading, but most of this benefit is observed under certain conditions, such as orthographic similarity or phonological consistency between the preview and target words (Tsai et al., 2004), or in the case of compound words (with phonetic radicals) (Luo et al., 2018).

Other studies using eye movement technology have discovered that phonological information plays a role in the later stage of lexical processing during Chinese reading. For example, Wong and Chen (1999) used eye movement techniques and had participants read short Chinese passages with spelling errors, and the participants were warned that the passages might contain errors. Their results showed a reliable orthographic effect in both the early and later stages; on the other hand, the role of phonological information in lexical processing was observed only in the later stage but not in early processing. This result suggests that orthographic information, rather than phonological information, has a dominant and early role in the lexical processing of Chinese reading. However, researchers have proposed that participants’ awareness of passage errors might affect the way they read (Feng et al., 2001). Therefore, Feng et al. used the same paradigm to further investigate the role of phonology and orthography in reading without informing the readers that spelling errors were included. The results showed that homophones only play a role in the later stage and have advantages over orthographically controlled words in the later stage. Generally, these studies all showed that phonological information is involved in the later stage of Chinese reading.

Nevertheless, previous studies either did not examine the role of orthography or phonology in reading under natural reading circumstances or failed to adequately separate orthographic similarity from homophone effects (without controlling orthographic similarity independently to demonstrate the role of phonology). For example, Feng et al. (2001) reported that homophones have advantages over orthographic controls in the later stage, whereas homophones also included orthographically similar and dissimilar words, and orthographic controls also included homophones and non-homophones. Therefore, it is unclear whether the role of phonological information in lexical access at a later stage in Chinese reading is also due to orthographic support, as it is in English reading. Further exploration of this problem is of particular significance to investigate the universality and unique properties of different script lexical access routes.

The present study was designed to determine the role and time course of orthographic and phonological processing in Chinese sentence reading using the eye movement technique, as well as to provide a reference for the universality or specificity of lexical access routes. As in previous studies, we addressed this issue by using the error disruption paradigm and manipulating three types of commonly misspelled characters (homographic errors, homophonic errors, and omitted stroke errors) of the initial target character in a sentence. Character misspelling is a common error that appears in written text, and research indicates that misspelled words interfere with lexical processing during normal reading (Kuperman et al., 2021). However, these words with spelling errors also provide insight into the mechanisms of lexical access in reading, which have significant implications for enriching eye movement theory in reading.

First, as in earlier research, we designed homophonic and homographic errors orthographically or phonologically similar to the correct forms (Wong and Chen, 1999; Feng et al., 2001). Our study, however, strictly controlled all homophones that were orthographically dissimilar to the target word (homographs that were also phonologically dissimilar to the target word) to clarify whether orthography is necessary to support the phonological effect observed at the late stage in Chinese reading. There is evidence that most misspelled characters also contain phonological or orthographic information, which might also influence lexical processing, and that different errors may affect lexical processing at early and late stages in different ways (Liu et al., 2014; Schotter et al., 2014). Therefore, by comparing the differences in eye movement reading patterns between homophonic and homographic errors, we investigated the role and time course of orthography and phonology in the Chinese processing of misspelled words. Second, different from previous studies, we also designed a third type of misspelled character, omitted stroke errors (Liu et al., 2014). It is common for some characters to appear with omitted strokes (not critical strokes) when they are written in Chinese characters. For example, the character心 (xin, meaning heart) can be written as 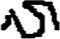 in handwriting. The new form 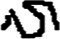 is a pseudo-character, but Chinese readers can easily recognize this form of error in written materials, even though it cannot be pronounced. Therefore, in addition to being similar to the target word in orthography, the omitted stroke error creates an unpronounceable character, allowing it to be used to investigate the effect of orthography on lexical processing with less interference from pronunciation. Moreover, the unpronounceable nature of omitted stroke errors allows us to compare them with homographic errors to examine the role of phonology in word processing during reading. Finally, we also designed a correct condition with the correct forms of the target characters as a baseline for comparing them with misspelled characters and examining the effect of spelling errors on reading.

By designing three types of commonly misspelled characters and using eye movement technology, the role of orthography and phonology in Chinese reading can be identified. The following assumptions can be made based on the above: First, if orthography plays a dominant role in lexical processing during Chinese reading, then homographic and omitted stroke errors will produce less interference than homophonic errors during early lexical processing, and homographic and omitted stroke errors should interfere equally during the early stage of lexical processing. Second, if phonology plays a role in lexical processing only under conditions of orthographic similarity in the later stage during Chinese reading, as in English, we should not observe a benefit from homophonic errors. Third, if there is a difference in interference with word processing between homographic and omitted stroke errors, it indicates that phonological information is activated in word processing.

## 2. Methods

### 2.1. Participants

Thirty-two young adults aged 18–26 (*M* = 20.21 years, *SD* = 1.90) from Tianjin Normal University were paid to participate in the reading experiment. All participants were native Chinese speakers with normal or corrected-to-normal vision (acuity values are better than 20/40, tested using a Snellen eye test).

### 2.2. Stimuli and design

**Figure 1:**
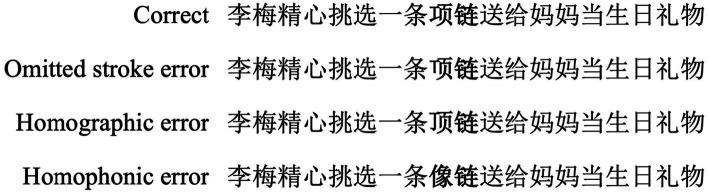
An example sentence in each condition. The sentence translated as “*Li Mei selected a necklace carefully as a birthday gift for her mother.*” The target region has been highlighted in bold but was presented normally in the actual experiment.

Sixty-four experimental sentence frames were constructed for the current study. Each sentence frame consisted of three types of target words with spelling errors, which form three misspelled conditions (omitted stroke error, homographic error, and homophonic error), and a correct target word as the correct condition. Example sentences can be seen in Figure 1.

In the correct condition, the first character (e.g., 项-xiang, meaning neck) of the target word (e.g., 项链, meaning necklace) was presented in the correct form so that participants could read these sentences naturally. In the omitted stroke error condition, a stroke was removed from the first characters of target words; then, the first characters became a pseudo-character (e.g., 项), but they looked like (orthographically similar) the characters in the correct condition (the target words in the omitted stroke error condition were not real words). In the homographic error condition, the initial character of the target word was replaced by another character with similar orthography and dissimilar phonology (e.g., 顶-ding, meaning peak), but a real word could not be formed with the second character (e.g., 链-lian, meaning chain). The final condition is the homophonic error condition, in which the first character of the target word was replaced by a homophonic character with dissimilar orthography (e.g., 像-xiang, meaning like); likewise, a real word cannot be formed with the second character (e.g., 链). The ANOVA analysis indicated that the frequency of the first characters in the correct condition (*M* = 230 counts/million), homographic error condition (*M* = 290 counts/million), and homophonic error condition (*M* = 260 counts/million) was not significantly different (*F* = 0.170, *p* = 0.844). Word frequency was based on the SUBTLEX-CH database (Cai and Brysbaert, 2010). Moreover, the stroke number of the first characters was matched (*F* = 0.617, *p* = 0.514) in the correct condition (*M* = 8.36), homographic error condition (*M* = 8.34), and homophonic error condition (*M* = 8.78).

Experimental sentences were 17–24 characters in length (*M* = 20 characters) and presented in a single line. The target word was neither in the first five characters’ positions of sentences nor in the last five characters’ positions. A cloze task was conducted with another 10 native Chinese speakers who were only provided with the beginning parts of the sentence before the target words and were asked to fill in the next acceptable word. The final results showed that target words in the experimental sentences were unpredictable in the sentence frames (predictability value, *M* = 2.2%). Additionally, naturalness ratings on a scale from 1 (very unnatural) to 7 (very natural) were obtained for each experimental sentence from 10 native Chinese speakers who did not participate in the cloze task or the actual experimental test on a scale from 1 (very unnatural) to 7 (very natural). The results showed that the experimental sentences were highly natural (*M* = 6.59).

All of the sentences were randomly sampled, so each participant saw a sentence containing either a correct target word or one of the misspelled words (a homographic error, omitted stroke error, or a homophonic error) only once, and there was an equal number of four conditions (16 sentences in each condition) for each participant. We constructed an equal number of experimental and filler sentences to prevent participants from predicting misspelled errors within the experimental sentences. In summary, each subject read 8 practice sentences at first, and then 64 experimental sentences and 64 filler sentences were presented randomly during the core experiment.

### 2.3. Apparatus and procedure

An SR EyeLink 1,000 plus eye-tracking system recorded each participant’s right-eye gaze location every millisecond during binocular viewing. This system has high spatial (<0.01°RMS) and temporal (1,000 Hz) resolution. Stimuli were presented in Song font as black text on a gray background (RGB: 220, 220, 220) at a viewing distance of 75 cm. Each character subtended approximately 0.70° horizontally, so characters were presented at a convenient size for reading.

Participants were asked to read normally to ensure that every sentence was comprehended. They were not told that some sentences would include errors. A 3-point horizontal calibration procedure was conducted across the same line in which the text was presented. The calibration accuracy was checked before the presentation of each trial and recalibrated as necessary (i.e., for calibration error <0.30°). At the start of each trial, a fixation square equal in size to one character was presented on the left side of the computer screen. Once the participants stably fixed the fixation square, a sentence was presented with the first character replacing the square. Participants needed to press a response key after they finished reading each sentence. The sentence was then replaced by a comprehension question in 33% of trials, and participants needed to respond by pressing a button. The whole experiment lasted approximately 30 min for each participant.

## 3. Results

Accuracy in answering comprehension questions was high for all participants (*M* = 98%), indicating that all the participants read carefully and understood the sentences very well. We removed the fixations less than 80 ms or longer than 1,200 ms (affecting 5.0% of fixations).

We mainly reported several word-level measures, which were as follows: first-pass reading measures (i.e., the initial processing of a word prior to a fixation to its right or skipping rate; Rayner, 2009), including the first fixation duration (FFD, the duration of the first fixation on the target word during the first-pass reading), single-fixation duration (SFD, the duration in which there is only one fixation on the target word during the first-pass reading), gaze duration (GD, the total time of all first-pass fixations on the target word), skipping rate (SR, the probability of the target word was not fixated on during the first-pass reading), and first-pass fixation count (FFC, the number of the first-pass fixations on the target word). We also examined regression path duration (RPD, the sum of all fixations from the first fixation on a word during the first-pass reading until the eyes move to the right of the word, including the time spent rereading the sentence before the target word), which refers to the difficulty in integrating the target word into the context of the sentence before moving forward (Liversedge et al., 1998). In addition, we reported total reading time (TRT, the sum of all fixations on the target word, including regressions) and total fixation count (TFC, the total number of all fixations on the target word) as measures of late lexical processing. Finally, we reported the sentence reading time (SRT, the time from the onset of a sentence display until the participants pressed the response key to indicate that they had finished reading) at the sentence level. Sentence reading time can reflect the influence of different misspelled characters on Chinese sentence reading.

The data were analyzed by the *lme*4 package (Bates et al., 2015) in the R environment (R Development Core Team, 2018), with participants and items included as crossed random effects. Dichotomous variables were analyzed using generalized linear models, and continuous variables were analyzed using linear mixed-effects models. For all analyses, a *t/z* value greater than 1.96 indicated statistical significance.

### 3.1. Target word-level analyses

The means and standard errors for word-level and sentence-level measures are shown in Table 1, and the analysis results of the linear mixed model are shown in Table 2.

**Table 1:** Means and standard errors for the target word-level and sentence-level measures.

	Correct	Omitted stroke error	Homographic error	Homophonic error
SFD (ms)	235 (4)	255 (6)	250 (6)	272 (7)
FFD (ms)	236 (4)	254 (6)	257 (5)	274 (6)
GD (ms)	269 (7)	316 (10)	336 (14)	370 (13)
RPD (ms)	314 (10)	388 (16)	408 (18)	456 (13)
TRT (ms)	336 (10)	415 (15)	553 (24)	603 (23)
FFC	1.14 (0.02)	1.25 (0.03)	1.25 (0.04)	1.34 (0.04)
TFC	1.26 (0.05)	1.44 (0.07)	1.85 (0.09)	2.07 (0.09)
SR	0.23 (0.02)	0.22 (0.02)	0.22 (0.02)	0.19 (0.02)
SRT (ms)	2,581 (50)	2,679 (56)	2,914 (62)	3,021(62)

**Table 2:** Results for the mixed-linear model analysis of target words measures and sentence reading times.

Measure	corr-omit			corr-graph			corr-phon			omit-graph			omit-phon			graph-phon		
	*b*	*SE*	*t/z*	*b*	*SE*	*t/z*	*b*	*SE*	*t/z*	*b*	*SE*	*t/z*	*b*	*SE*	*t/z*	*b*	*SE*	*t/z*
SFD	20.01	7.67	2.61^**^	18.54	7.65	2.42^*^	41.65	7.70	5.41^***^	−1.47	7.75	−0.19	21.64	7.82	2.77^**^	23.11	7.79	2.97^**^
FFD	17.81	7.28	2.45^*^	20.57	7.30	2.82^**^	37.82	7.21	5.24^***^	2.76	7.24	0.38	20.01	7.17	2.79^**^	17.25	7.19	2.40^*^
GD	46.20	14.87	3.11^**^	67.79	14.91	4.55^***^	103.19	14.74	7.00^***^	21.59	14.79	1.46	56.99	14.65	3.89^***^	35.39	14.70	2.41^*^
RPD	72.76	21.22	3.43^***^	97.28	21.27	4.57^***^	145.51	21.03	6.92^***^	24.52	21.11	1.16	72.75	20.90	3.48^***^	48.23	20.97	2.30^*^
TRT	80.70	23.84	3.39^***^	218.03	23.92	9.12^***^	278.15	23.55	11.81^***^	137.33	23.70	5.79^***^	197.45	23.42	8.43^***^	60.12	23.49	2.56^*^
FFC	0.11	0.04	2.59^**^	0.12	0.04	2.75^**^	0.21	0.04	5.06^***^	0.01	0.04	0.17	0.10	0.04	2.46^*^	0.09	0.04	2.28^*^
TFC	0.19	0.07	2.56^*^	0.59	0.07	8.06^***^	0.82	0.07	11.34^***^	0.40	0.07	5.52^***^	0.64	0.07	8.79^***^	0.24	0.07	3.25^**^
SR	−0.08	0.16	0.63	−0.06	0.16	0.71	−0.24	0.17	0.15	0.02	0.17	0.91	−0.16	0.17	0.34	−0.18	0.17	0.29
SRT	112.01	59.53	1.88	342.98	59.69	5.75^***^	471.11	59.48	7.92^***^	230.97	59.52	3.88^***^	359.11	59.39	6.05^***^	128.14	59.45	2.16^*^

*p < 0.05, **p < 0.01, ***p < 0.001. corr, correct; omit, omitted stroke error; graph, homographic error; phon, homophonic error.

**Figure 2:**
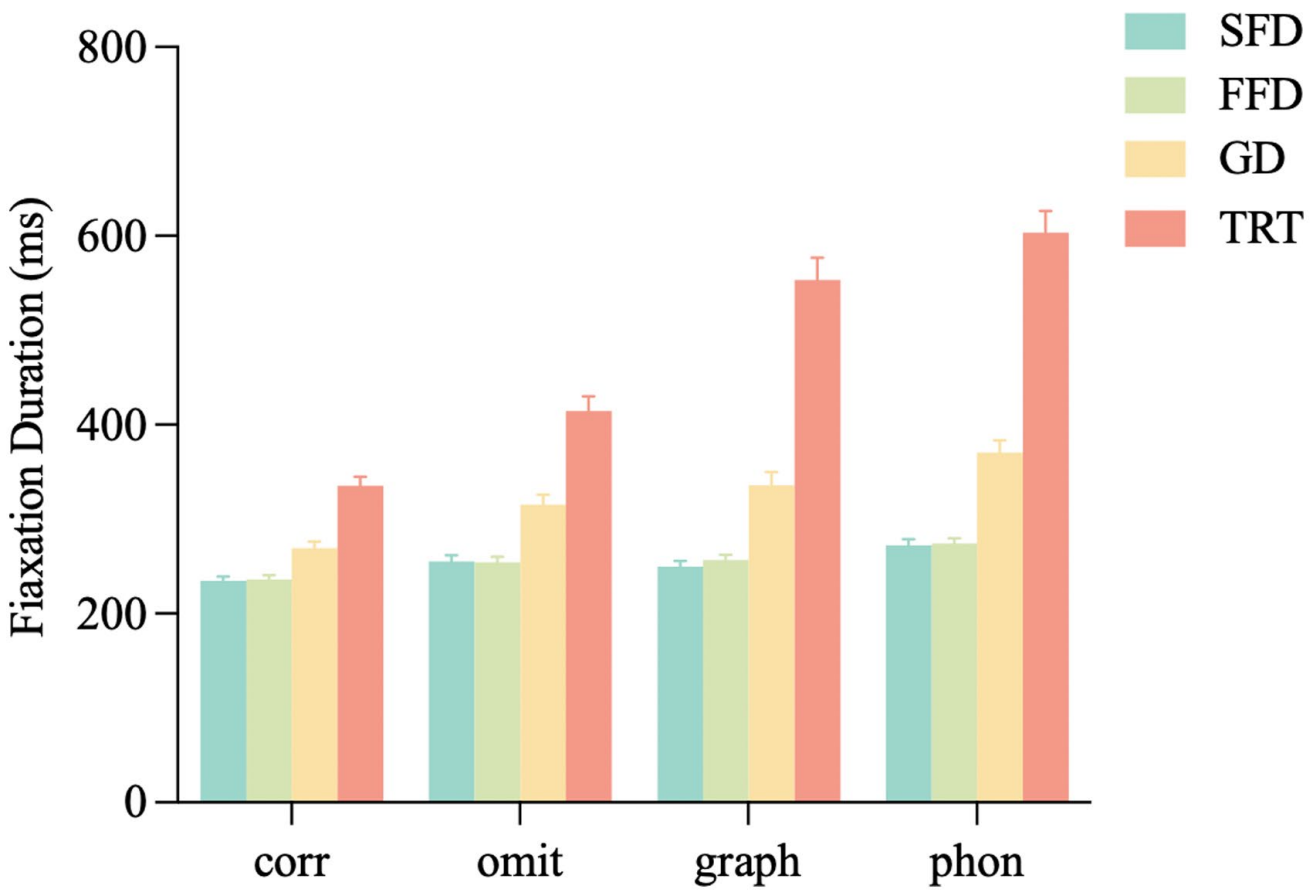
Fixation durations on the target word under different conditions. SFD, single fixation duration; FFD, first fixation duration; GD, gaze duration; TRT, total reading time; corr, correct; omit, omitted stroke error; graph, homographic error; phon, homophonic error. Error bars show the standard error of the mean.

*Single-fixation duration, first fixation duration, and gaze duration*: The single-fixation duration, first fixation duration, and gaze duration showed the same pattern, as shown in Figure 2. Three duration measures in the homophonic error condition were significantly longer than those in the homographic error (*t*s > 2.40, *p*s < 0.05), omitted stroke error (*t*s > 2.77, *p*s < 0.01), and correct conditions (*t*s > 5.24, *p*s < 0.001). Compared to the correct condition, the fixation durations were longer in the homographic error (*t*s > 2.42, *p*s < 0.05) and omitted stroke error (*t*s > 2.45, *p*s < 0.05) conditions. No significant differences were found in the homographic error condition versus the omitted stroke error condition in these three measures (|*t*|s < 1.46, *p*s > 0.05).

*Regression path duration*: Compared to the correct condition, the regression-path duration was longer in the homophonic error (*t =* 6.92, *p* < 0.001), homographic error (*t =* 4.57, *p* < 0.001) and omitted stroke error (*t =* 3.43, *p* < 0.001) conditions. Moreover, the regression-path duration was significantly longer in the homophonic error condition than in the homographic error (*t =* 2.30, *p* = 0.02) and omitted stroke error (*t =* 3.48, *p* < 0.001) conditions. The difference in the regression-path duration between the homographic error condition and the omitted stroke error condition did not reach a significant level (*t =* 1.16, *p* = 0.25).

*Total reading time*: As shown in Figure 2, compared to the correct condition, the total reading time was longer in the homophonic error (*t =* 11.81, *p* < 0.001), homographic error (*t =* 9.12, *p* < 0.001) and omitted stroke error (*t =* 3.39, *p* < 0.001) conditions, in which the homophonic error condition was significantly longer than the homographic error (*t =* 2.56, *p* = 0.01) and omitted stroke error (*t =* 8.43, *p* < 0.001) conditions. In addition, the total reading time in the homographic error condition was significantly longer than that in the omitted stroke error condition (*t =* 5.79, *p* < 0.001).

**Figure 3:**
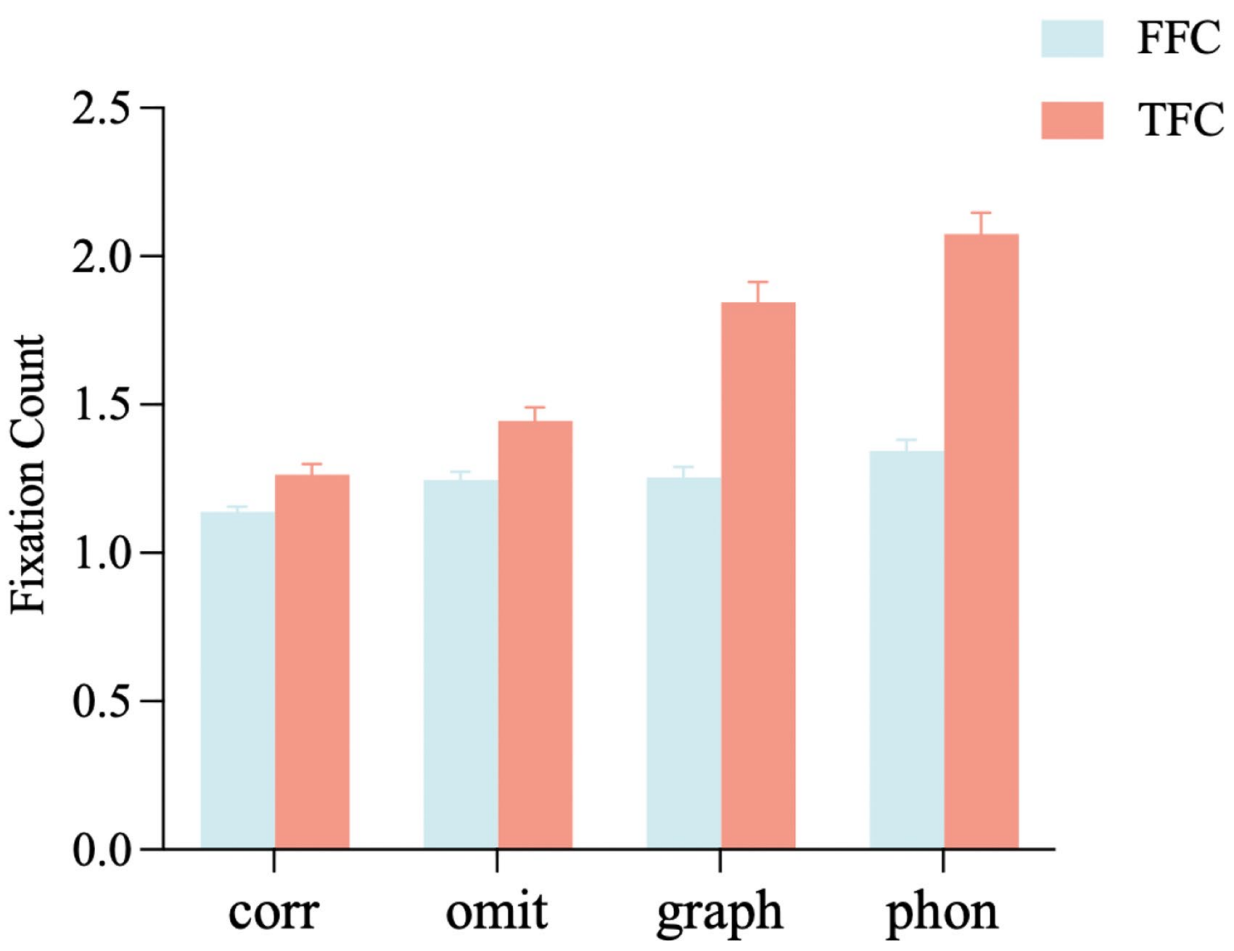
Fixation count on the target word under different conditions. FFC, first-pass fixation count; TFC, total Fixation count; corr, correct; omit, omitted stroke error; graph, homographic error; phon, homophonic error. Error bars show the standard error of the mean.

*First-pass fixation count*: As shown in Figure 3, the homophonic error condition caused more fixations than the homographic error (*t =* 2.28, *p* = 0.02), omitted stroke error (*t =* 2.46, *p* = 0.01), and correct (*t =* 5.06, *p* < 0.001) conditions during the first-pass reading. Compared to the correct condition, there were significantly more fixations in the homographic error (*t =* 2.75, *p* = 0.01) and omitted stroke error (*t =* 2.59, *p* = 0.01) conditions. No significant differences were found between the homographic error and omitted stroke error conditions (*t =* 0.17, *p* = 0.87).

*Total fixation count*: For total fixation count, we found that compared to the correct condition, there were significantly more fixations in the homophonic error (*t =* 11.34, *p* < 0.001), homographic error (*t =* 8.06, *p* < 0.001), and omitted stroke error (*t =* 2.56, *p* = 0.01) conditions, in which the homophonic error condition caused significantly more fixations than the homographic error (*t =* 3.25, *p* = 0.01) and omitted stroke error (*t =* 8.79, *p* < 0.001) conditions. In addition, the total fixation count in the homographic error condition was significantly higher than that in the omitted stroke error condition (*t =* 5.52, *p* < 0.001).

*Skipping rate*: No effects of misspelled words were observed on the skipping rate measure (all |*z*|s < 1.43), and the skipping rate in all conditions was no more than 23%.

### 3.2. Sentence-level analyses

*Sentence reading time*: The sentence reading time in the homophonic error condition was significantly longer than that in the homographic error (*t =* 2.16, *p* = 0.03), omitted stroke error(*t =* 6.05, *p* < 0.001), and correct (*t =* 7.92, *p* < 0.001) conditions, and the sentence reading time in the homographic error condition was significantly longer than that in the omitted stroke error (*t =* 3.88, *p* < 0.001) and correct (*t =* 5.75, *p* < 0.001) conditions. There was a marginal significance for the sentence reading time results in the omitted stroke error and correct conditions (*t =* 1.88, *p* = 0.06).

## 4. Discussion

The present study was conducted mainly to investigate the relative role of orthographic and phonological processing during reading Chinese sentences by using the error disruption paradigm (Daneman and Reingold, 1993). By comparing the patterns of disruptions in eye movements caused by different types of misspelled characters, we found that spelling errors could disrupt lexical processing in sentence reading. However, the extent of disruption varied in different errors encountered by readers. Specifically, our results showed that the homophonic error condition caused more and longer fixations than the homographic error and omitted stroke error conditions in both the earlier and later stages of lexical processing; moreover, the omitted stroke error condition caused less interference (shorter total reading time) than the homographic error condition in the later stage. This pattern of results suggests that orthographic information plays a dominant role in lexical processing during Chinese reading, while phonological information plays a limited role at the later stage.

Our study found that homographic and omitted stroke errors are less disruptive than homophonic errors; homographic and omitted stroke errors produced identical disruptions for the first-pass reading and regression path times. This result supports the first hypothesis that orthographic processing plays a dominant role in the lexical processing of Chinese reading rather than phonological processing (Zhou and Marslen-Wilson, 1996; Zhou et al., 1999a; Zhang et al., 2020), supporting the direct access model. Interestingly, our findings in reading Chinese sentences are consistent with those obtained in word identification studies that use isolated characters or two-character Chinese words (Chen et al., 1995; Zhou et al., 1999b). Nevertheless, our findings were inconsistent with those in English studies that phonology plays a dominant role in the early stage of reading processing (Pollatsek et al., 1992; Sereno and Rayner, 1992). Different from alphabetic writing systems, Chinese is a kind of logographic writing system, and characters can represent semantic information more directly (Li et al., 2022). Previous studies have shown that under certain conditions (e.g., orthographically similar; Tsai et al., 2004), phonological information can be activated in the early stage of word processing (Vasilev et al., 2019). As far as our study is concerned, orthography, rather than phonology, plays an important role in the early stages of word processing when homophones and target words are not orthographically similar.

Our results found that omitted stroke errors were less costly (compared to the correct condition) and that the effect of homographic errors is larger, which is not surprising since participants can easily “normalize” visually similar characters. This is consistent with the Chinese reading model (CRM, Li and Pollatsek, 2020), which assumes that the reader’s bottom-up identification of Chinese characters is achieved through template matching. Examples include comparing the input Chinese character image with the Chinese character template represented in Chinese character units and identifying the to-be-recognized image (orthographic representation) as the closest matching object (target word). Combined with our findings, orthography plays an important role in word processing in Chinese reading. Similarly, researchers found that replacing the beginning, middle, or end letters of the target words with visually similar letters took less reading time than using dissimilar letters (Rayner and Kaiser, 1975). Other studies also showed that letter-by-number replacements lead to more reading costs than letter-by-symbol replacements (especially when the substituted letters are at the beginning of the word) (Duñabeitia et al., 2009). However, a Spanish study found that the interaction effect between the “feature” and “letter” levels in visual word recognition models is also limited by the function of diacritical marks in the language (Marcet et al., 2020). Overall, these studies suggest that orthographic processing is particularly important for visual word recognition.

The results of our study had two significant findings at the later stage of lexical processing and reading (in total fixation count and total reading time). First, homophone errors were the most destructive, followed by homograph and missed stroke errors. The results of this study were inconsistent with previous studies, which reported that phonology plays a role in the later stage of lexical processing during reading (Wong and Chen, 1999; Feng et al., 2001). This suggests that in Chinese reading, when the target words and homophones are not visually similar, the facilitation effect of phonology in the later stage is eliminated, which is consistent with studies of English reading (Daneman and Reingold, 1993; Daneman et al., 1995). Second, our study found that compared with equal disruption in the early processing stage, homograph errors disrupted the total reading time more than omitted stroke errors. This result suggested that in the later stage of word processing, implausible phonological information from orthographically similar characters may begin to interfere with lexical processing. It should be noted that we cannot exclude the possibility that homophones and homographs may have semantic interference at the late stage of reading processing. However, at least our research shows that homophone errors interfere more with total reading time than non-homophone errors, suggesting that phonology has a weaker and more limited role in the later stage of lexical processing without orthographic similarity.

On the other hand, another important measure in Chinese sentence reading is sentence reading time, which can reflect the relative impact of different misspelled characters on sentence reading performance. The results showed that homophonic errors caused more disruption than homographic errors and missed stroke errors, and removing a stroke had a marginally significant effect on sentence reading. This indicated that phonological character errors strongly impair the entire sentence reading performance, and the removal of a stroke has a minimal effect on sentence reading. Combined with the results based on target word analysis, it was found that the omission of a stroke interfered with Chinese character recognition or text processing, allowing readers to easily guess the correct characters from the text (Liu et al., 2014). Li et al. (2009) argued that Chinese word segmentation and recognition must rely on top-down processing. In combination with the simulation results of the CRM (Li and Pollatsek, 2020), it is found that all Chinese characters in the perceptual span are activated in parallel (similar to the SWIFT model, Engbert et al., 2005). The activated words include all words that may be composed of activated Chinese characters, competing for the only winner. If a word is orthographically similar to the target word (visual similarity), the higher the degree of activation of lexical nodes, the easier it is to identify. Therefore, the information on Chinese characters and the existing lexical representation of readers are particularly important for lexical access.

Our study has some implications for constructing models of lexical access in reading. Our findings showed that orthographic information plays a relatively important role in the early stages. In line with English studies (Daneman and Reingold, 1993; Daneman et al., 1995), we did not find the homophone facilitation effect in the later stage when the correct word and homophones were not orthographically similar. The direct access hypothesis (Zhou and Marslen-Wilson, 1996; Zhou et al., 1999a) is more consistent with the early stage of lexical processing in Chinese reading. It should be noted that although we emphasize the dominant role of orthographic processing during reading, this does not mean that we completely neglect the role of phonological processing in Chinese reading. Our study found that phonological processing also involves the late stage of word processing; it is simply too slow and weak to affect the early stage of processing. Our results are consistent with the ERP research (Liu et al., 2011), suggesting that the direct access and dual-rote hypothesis may well explain the mechanism of word processing during reading. More research is needed on the role of phonological and orthographic processing in lexical access by electrophysiological techniques with higher temporal and spatial resolution. Additionally, factors such as word frequency (Wang et al., 2021), reading ability (e.g., college students with dyslexia show delayed phonological activation; Denis-Noël et al., 2020), and reading mode (e.g., online activation of orthography in phonological representation in spoken language; Perre et al., 2009) can be considered to systematically construct the cognitive mechanisms of lexical access in reading.

It is worth mentioning that our study has applied implications. Consistent with previous studies (Liu et al., 2014; Kuperman et al., 2021), our study shows that misspelled characters negatively affect word recognition and reading comprehension. Therefore, improving the writing quality (correct spelling) of words is particularly critical for fluency in reading. Especially in the education and teaching fields, using correct spelling and forming accurate orthographic representations is important for improving children’s word recognition and reading abilities (Siegelman et al., 2020; Wegener et al., 2020). In addition, our study found that removing only one stroke had little effect on sentence context reading. According to Tsao and Wang (1983), only the upper half part of the characters was easier to identify than the other three parts (lower, right, and left). Future research can further explore the effect of simplified parts of Chinese characters on word recognition in sentence context reading and contribute to the simplification process of Chinese characters.

In conclusion, the results of the present study mainly examined the relative contributions of orthographic and phonological activation of lexical access during reading Chinese sentences. We found evidence that orthography plays a dominant role in the early stage of Chinese reading, whereas phonology plays a weaker and more limited role in the later stage. Our findings suggest that the direct access and dual-rote hypothesis may well explain the mechanism of lexical access in Chinese reading. Our results also support the reading model in Chinese proposed by Li and Pollatsek (2020).

## Data availability statement

The datasets presented in this study can be found in online repositories. The names of the repository/repositories and accession number(s) can be found at: https://doi.org/10.6084/m9.figshare.21930702.v1.

## Ethics statement

The studies involving human participants were reviewed and approved by Tianjin Normal University. The patients/participants provided their written informed consent to participate in this study.

## Author contributions

ZL, ZY, LZ, LL, LS, and WJ participated in the design of the experiment. ZL and ZY collected and analyzed the data. The draft manuscript was prepared by ZL and ZY, and it was revised by WJ, ZL, and LZ. LL and LS provided good suggestions. All authors contributed to the article and approved the submitted version.

## Funding

This research was supported by a grant from the National Natural Science Foundation of China (32271119) and the Fujian Social Science Planning Project under grant (FJ2020C071).

## Conflict of interest

The authors declare that the research was conducted in the absence of any commercial or financial relationships that could be construed as a potential conflict of interest.

## Publisher’s note

All claims expressed in this article are solely those of the authors and do not necessarily represent those of their affiliated organizations, or those of the publisher, the editors and the reviewers. Any product that may be evaluated in this article, or claim that may be made by its manufacturer, is not guaranteed or endorsed by the publisher.
